# LINC00665 Targets miR-214-3p/MAPK1 Axis to Accelerate Hepatocellular Carcinoma Growth and Warburg Effect

**DOI:** 10.1155/2021/9046798

**Published:** 2021-11-10

**Authors:** Hongyu Wan, Yi Tian, Juan Zhao, Xiao Su

**Affiliations:** Department of Gastroenterrology, Minhang Branch, Zhongshan Hospital, Fudan University, Shanghai 201100, China

## Abstract

Inhibition of aerobic glycolysis is a hopeful method for cancer treatment. In this study, we aimed to explore LINC00665/miR-214-3p/MAPK1 role in regulating cell viability and aerobic glycolysis in hepatocellular carcinoma (HCC). The expressions of LINC00665 in 50 paired HCC tissues and normal tissues were determined by qRT-PCR. Pearson analysis was applied to evaluate the association between the expression levels of miR-214-3p, LINC00665, and MAPK1 in HCC tissues. The interactions between miR-214-3p and LINC00665 or MAPK1 were determined by luciferase reporter assay and RNA immunoprecipitation. CCK-8 and colony formation assays were used for cell viability evaluation. Lactate production, glucose consumption, and ATP levels were measured to assess Warburg effect. The results showed that LINC00665 was overexpressed in HCC, which positively associated with MAPK1 level and negatively associated with miR-214-3p level in HCC tissues. Overexpression of LINC00665 led to significant enhancements in cell viability and colony formation, whereas this effect was weakened when miR-214-3p was overexpressed or MAPK1 was downregulated. In addition, deletion of LINC00665 expression repressed tumor formation *in vivo*. Mechanically, LINC00665 increased MAPK1 expression through binding to miR-214-3p. Collectively, this study revealed that LINC00665 accelerated cell growth and Warburg effect through sponging miR-214-3p to increase MAPK1 expression in HCC.

## 1. Introduction

Hepatocellular carcinoma (HCC) is one of the most common malignancies [1]. Most patients with HCC show a dismal prognosis due to the high rates of metastasis and recurrence [2]. Due to the difficulties in early diagnosis and the absence of effective treatment methods, the 5-year survival rate of HCC remains less than 20% [3]. Therefore, it is essential to further explore the mechanism underlying HCC occurrence and development.

Aerobic glycolysis, also known as “Warburg effect,” is a trait of cancer cells. Cancer cells obtain energy to maintain the rapid growth through increasing of the uptake and consumption of glucose and enhancing glycolytic activity and excessive lactate secretion even in the presence of high oxygen concentration [4]. Evidence has demonstrated that Warburg effect plays crucial roles in accelerating cancer cell growth, migration, and drug resistance [5,6]. Inhibition of aerobic glycolysis is of hope for oncotherapy including HCC [7,8 ].

Long noncoding RNAs (lncRNAs) are a category of noncoding RNAs which are ≥200 bp in length. About 50,000 lncRNAs have been identified, but only a few lncRNAs have been investigated [9]. More and more researchers have demonstrated that lncRNAs are often dysregulated in cancers and serve as both oncogenes and suppressors in various kinds of cancers [10, 11]. One of the mechanisms by which lncRNAs are implicated in tumorigenesis is acting as competing endogenous RNAs (ceRNA) of microRNA (miRNA) and thereby facilitating the transcription of target mRNAs [12, 13]. Up to now, long intergenic non-protein-coding RNA 665 (LINC00665) has been found to show a higher expression pattern in cancers and to be closely implicated in carcinogenesis, including breast cancer [14], prostate cancer [15], gastric cancer [16], colorectal cancer [17], and ovarian cancer [18]. Shan et al. [19] found that LINC00665 level was increased in HCC tissues and cells and associated with shorter overall survival, and depletion of LINC00665 repressed cell viability and promoted cell apoptosis and autophagy in HCC via the miR-186-5p/MAP4K3 axis. However, the role of LINC00665 in aerobic glycolysis and the underlying mechanism still remain largely ignorant.

miR-214-3p expression was reported to be decreased in primary HCC samples when compared to normal liver tissues, and miR-214-3p upregulation suppressed cell proliferation and resulted in cell G1 phase arrest in HCC HepG2 and HUH-7 cells [20]. Hao et al. [21] reported that miR-214-3p could target mitogen-activated protein kinase 1 (MAPK1) in renal cell carcinoma. In the current study, we found that miR-214-3p had putative binding sites with LINC00665, indicating that the LINC00665/miR-214-3p/MAPK1 axis might take part in HCC progression. Thus, the current study was performed to reveal whether LINC00665 regulates aerobic glycolysis and cancer growth via sponging of miR-214-3p to increase MAPK1 expression in HCC.

## 2. Materials and Methods

### 2.1. Patient Tissue Samples

Fifty HCC and the adjacent noncancer tissues were obtained from patients with HCC between March 2013 and March 2015. The written informed consents were signed by patients or their parents. We have gotten the approval for this study from the Ethics Committee of Minhang Branch, Zhongshan Hospital, Fudan University, before carrying it out.

### 2.2. Bioinformatics Analysis

The starBase database (https://starbase.sysu.edu.cn/) was applied to analyze the expression levels of LINC00665, miR-214-3p, and MAPK1 in HCC samples and LINC00665 association with patients' overall survival, as well as to predict the binding sites between miR-214-3p and LINC00665/MAPK1.

### 2.3. Cell Culture Condition

The four human HCC cell lines SNU-387, SNU-423, SNU-449, and SNU-398 were acquired from American Type Culture Collection (ATCC, VA, USA) and kept in RPMI-1640 Medium with 10% fetal bovine serum (FBS) and 1% (v/v) penicillin-streptomycin as supplementations. All cells were placed at 37°C in an atmosphere containing 5% CO_2_. Regents used in this study were all brought from Thermo Fisher Scientific (USA).

### 2.4. Lentivirus Infection

A total of 2 × 10^5^ cells were seeded into every well of the 6-well plates and cultured at 37°C overnight. On the next day, the cells were infected with sh-LINC00665, lentiv-LINC00665, mimic-miR-214-3p, and sh-MAPK1-1/-2 (GenePharma, Shanghai, China) to modulate LINC00665/miR-214-3p/MAPK1 expression with the help of polybrene (6 *μ*g/ml). Lentiv-NC, sh-NC, and mimic-NC were used as negative controls. The infected cells were cultured in RPMI-1640 Medium with puromycin (6 *μ*g/ml) for fourteen consecutive days to construct the stably transfected cells that were used in the clone formation assay or animal assay.

### 2.5. Quantitative Reverse Transcription-PCR (qRT-PCR)

Total RNA was isolated using TRIzol reagent (Invitrogen, USA) and then subjected to cDNA synthesis with the help of PrimeScript RT Master Mix Kit (RR036A; Takara). Next, the cDNA was applied to PCRs detection with 2 × SYBR Green PCR Master Mix (Solarbio, Beijing, China) in 7500 Real-Time PCR System (Applied Biosystems, USA). To detect miR-214-3p expression, TaqMan™ Advanced miRNA cDNA Synthesis Kit (Thermo Fisher Scientific, MA, USA) and TaqMan™ MicroRNA assay (Applied Biosystems, USA) were used, with U6 as an internal control. The reactions were as follows: 95°C for 1 min and 39 cycles including 95°C for 15 s and 60°C for 1 min. The relative expression of mRNA was calculated based on the 2^−∆∆Cq^ method [22]. Primers are shown in Table 1.

### 2.6. Western Blotting

Total protein samples were obtained using lysis buffer (Solarbio, Beijing, China) supplemented with 1% protease inhibitor (Solarbio). After centrifugation at 4°C for 30 min, Bicinchoninic Acid Protein Assay kits (Thermo Fisher Scientific) were applied to examine protein concentrations in the light of specifications. Nuclear and cytoplasmic proteins were obtained with the help of the Nuclear Protein Extraction Kit (Solaibio) in accordance with the manufacturer's descriptions. Then, protein samples were loaded to 10% SDS-polyacrylamide gel and submitted to electrophoresis and transformation to polyvinylidene difluoride membranes (PVDF; Millipore, Billerica, MA, USA). Next, the membranes were probed with first antibodies overnight at 4°C after being blocked in 5% nonfat milk for 1 hour at room temperature: anti-*β*-actin antibody (1 : 5000 dilution; cat. no. ab8226, Abcam, MA, USA), p-p38 MAPK (1 : 1000 dilution; cat. no. #4511, Cell Signaling Technology, MA, USA), and anti-MAPK1 antibody (1 : 3000 dilution; cat. no. ab182453, Abcam). Then, the membranes were probed with the HRP-conjugated secondary antibodies at room temperature for 1 hour. Following incubation with ECL reagent (Millipore, USA), the protein signaling was measured by using ProfiBlot 48 (Tecan, Switzerland) and quantified by using ImageJ v2.1.4.7 (National Institutes of Health, Bethesda, MD, USA).

### 2.7. Double Luciferase Gene Reporter Measurement

The wild-type (WT) or mutated (Mut) luciferase vectors of MAPK1/LINC00665 were produced by GenePharma (Shanghai, China). Cells were transfected with mimic-NC or mimic-miR-214-3p and WT or MUT vector of MAPK1/LINC00665 together using Lipofectamine™2000 (Thermo Fisher Scientific). Following 48 hours of infection/transfection, each group of cells was harvested for analysis of the luciferase activity according to the Dual Luciferase Reporter Assay protocol (Promega, Madison, WI, USA).

### 2.8. RNA Immunoprecipitation (RIP)

The interaction of miR-214-3p and LINC00665/MAPK1 was evaluated using RIP technology as previously described [23] with a Magna RIP kit (Millipore) according to the manufacturer's protocols. Cell lysate was incubated with magnetic beads coated anti-Ago2 antibody (Abcam) or anti-IgG antibody (Abcam). After that, qRT-PCR assay was performed to detect the expression levels of miR-214-3p or LINC00665 or MAPK1 analysis in the complex.

### 2.9. CCK-8 Assay

Three thousand breast cancer cells were placed into each well of the 96-well plates. Cell transfection was then executed following cell adherence. Then, the culture medium was replaced with 10 *μ*L CCK-8 solution (Abcam) which was used for cell viability detection. Three hours after incubation with CCK-8 solution at 37°C, the OD values (450 nm) were examined by a spectrophotometer (Thermo Fisher Scientific).

### 2.10. Colony Formation Assay

The stable cells were suspended at a density of 500 cells/100 *μ*l and 100 *μ*l suspension was added to every well of the 6-well plates which was replenished to 2 ml. Fourteen days after, the cells were fixed with 4% paraformaldehyde and stained with 0.1% crystal violet (Solarbio, Beijing, China) for 15 min at room temperature.

### 2.11. Wound Healing Assay

HCC cells were seeded into 24‐well plates at a density of 4 × 10^5^ cells/well and cultured in FBS-free medium. A 10 *μ*L sterile pipette tip was applied to scratch the cell layer, and the floating cells were removed by washing with PBS. The gap was imaged under a magnification of 40×.

### 2.12. Transwell Chambers

Chemotactic invasion of cells was evaluated using the Transwell chambers (8.0 *μ*m pore membranes, Corning, USA) precoated with 50 *μ*L Matrigel (BD Biosciences). Twenty-four hours following transfection, cells were plated in the upper chamber at a density of 10^5^ cells/well and cultured in serum-free medium. Cell culture medium containing 10% FBS was added to the bottom chamber. After incubation at 37°C for 24 hours, the invaded cells were fixed with 4% formaldehyde and stained with crystal violet. Cell numbers in five randomly selected fields were counted under a microscope.

### 2.13. Measurement of Lactate Production, Glucose Consumption, and ATP Levels

HCC cells (1 × 10^5^/well) were seeded into each well of 6-well plates and maintained at 37°C for 24 hours. Then, lactate production and glucose consumption from the medium sample were measured using commercial kits (cat. no. A019, Nanjing Jiancheng Biotech, Jiangsu, China; cat. no. E1010, Applygen Co., LTD., Beijing, China). ATP levels were examined using an ATP Colorimetric/Fluorometric Assay Kit (Sigma-Aldrich).

### 2.14. Animal Experiments

HCC cells (5 × 10^5^) stably transfected with sh-NC/sh-LINC00665 were injected into the armpit of 6-week male BALB/c nude mice (Charles River Laboratories Research Model Service, Beijing, China). Each group included five mice. Four weeks later, mice were euthanized by cervical dislocation, and the tumors were taken out and weighted. Tumor volume was estimated by V = length × width^2^/2. The animal study was done in Minhang Branch, Zhongshan Hospital, Fudan University, and approved by the Animal Care Committee of Minhang Branch, Zhongshan Hospital, Fudan University.

### 2.15. Statistical Analysis

Three independent experiments were carried out in the current study. Statistical analysis was performed by using SPSS (version 23.0). Student's *t*-test and one-way ANOVA followed by Tukey's tests were used for data analysis among two and multiple groups, respectively. Kaplan-Meier curves with log-rank tests were used to analyze the value of LINC00665 in predicting patients' overall survival time in HCC. Pearson analysis was applied to evaluate the association between the expression levels of miR-214-3p, LINC00665, and MAPK1 in HCC tissues. *p* < 0.05 indicated statistical significance.

## 3. Results

### 3.1. LINC00665 Expression is Elevated in HCC and Associates with Poor Prognosis

**Figure 1:**
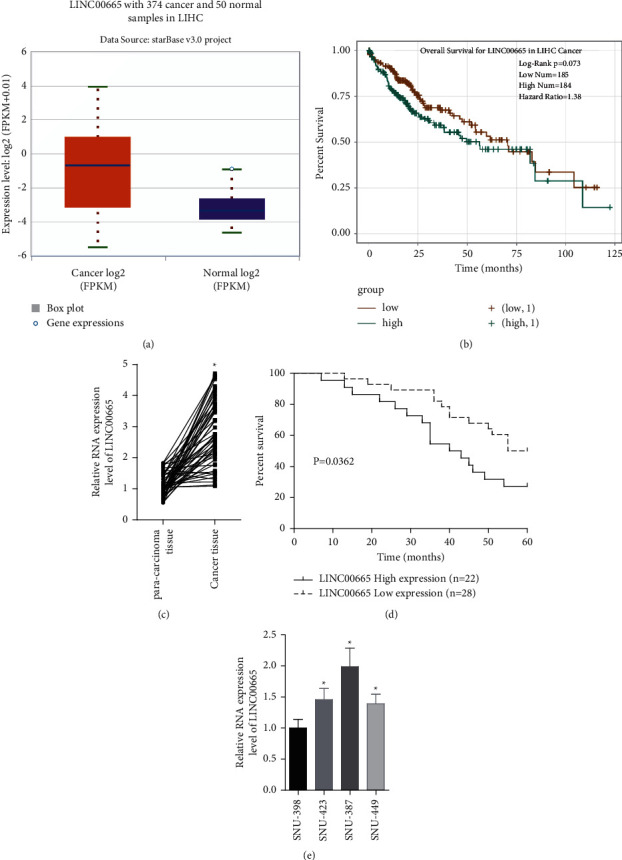
LINC00665 expression was elevated in HCC and associated with poor prognosis. (a) starBase database showed that LINC00665 expression in HCC tissues was elevated. (b) starBase database showed that high expression of LINC00665 predicted poor prognosis. (c) LINC00665 levels in 50 paired HCC tissues and para-carcinoma normal tissues were measured using qRT-PCR assay. (d) Kaplan-Meier curves were applied to analyze LINC00665 value in predicting patients' overall survival in HCC. (e) LINC00665 levels in SNU-387, SNU-423, SNU-449, and SNU-398 were measured by qRT-PCR assay (^∗^*P* < 0.05).

First, we assessed the expression and clinical significance of LINC00665 using the starBase software. LINC00665 level in HCC samples was elevated as compared with the normal samples (Figure 1(a)); and patients with LINC00665 high expression showed a shorter overall survival than those with LINC00665 low expression (Figure 1(b)). To verify it, 50 HCC tissues and the matched normal tissues were included. Consistently, the results from qRT-PCR and Kaplan-Meier curves showed that LINC00665 level was significantly increased in HCC tissues, which was predicted with a shorter overall survival (Figures 1(c) and 1(d)).  Figure 1(e) shows the expression levels of LINC00665 in four HCC cell lines (SNU-387, SNU-423, SNU-449, and SNU-398). As LINC00665 showed medium expression levels in SNU-423 and SNU-449 among the 4 cell lines, SNU-423 and SNU-449 cells were chosen for the following studies. These discoveries demonstrated that LINC00665 was upregulated in HCC and associated with poor prognosis.

### 3.2. LINC00665 Promotes HCC Viability and Aerobic Glycolysis

**Figure 2:**
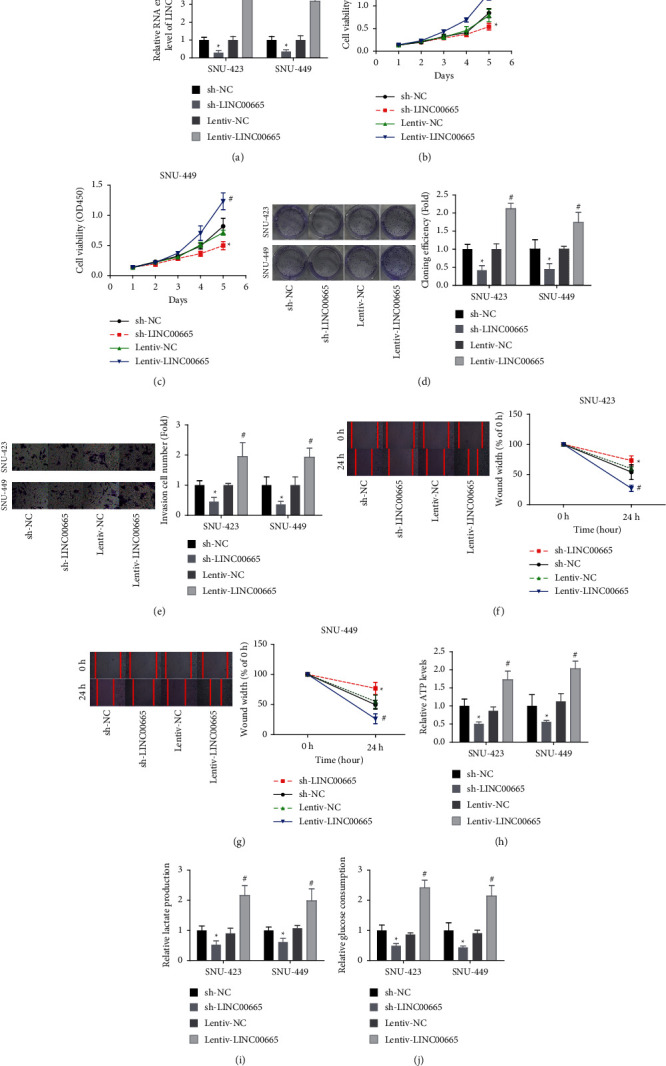
LINC00665 enhanced HCC viability and aerobic glycolysis. (a) The transfection efficiencies of sh-LINC00665 and lentiv-LINC00665 were detected by qRT-PCR assay. (b–d) LINC00665 effect on cell viability was determined using CCK-8 and colony formation assay. (e–g) LINC00665 role in regulating cell invasion and migration was assessed by Transwell chambers and wound healing assay. (h–j) The ATP levels, lactate section, and glucose consumption were tested in SNU-449 and SNU-423 cells following sh-LINC00665 or lentiv-LINC00665 infection (*n* = 3, *∗p* < 0.05 versus sh-NC group; #*p* < 0.05 versus lentiv-NC group).

Next, the gain- and loss-of-function assays were executed to reveal LINC00665 function in cell growth and aerobic glycolysis in HCC. LINC00665 level was significantly increased when cells were infected with lentiv-LINC00665, while it declined following cell infection with sh-LINC00665 (Figure 2(a)). Upregulation of LINC00665 markedly enhanced cell viability and colony formation abilities when compared with the control group in SNU-423 and SNU-449 cells, and silencing of LINC00665 caused the opposite result (Figures 2(b)–2(d)). In addition, LINC00665 overexpression significantly enhanced the invasiveness (Figure 2(e)) and migration ability (Figures 2(f) and 2(g)) of SNU-423 and SNU-449 cells. Moreover, the upregulation of LINC00665 led to obvious increases in ATP levels, lactate section, and glucose consumption in SNU-423 and SNU-449 cells, while knockdown of LINC00665 decreased the glucose consumption, lactate section, and ATP levels as compared with the control group (Figures 2(h)–2(j)). These results illustrated that LINC00665 promoted cell viability and aerobic glycolysis in HCC.

### 3.3. LINC00665 Targets miR-214-3p

**Figure 3:**
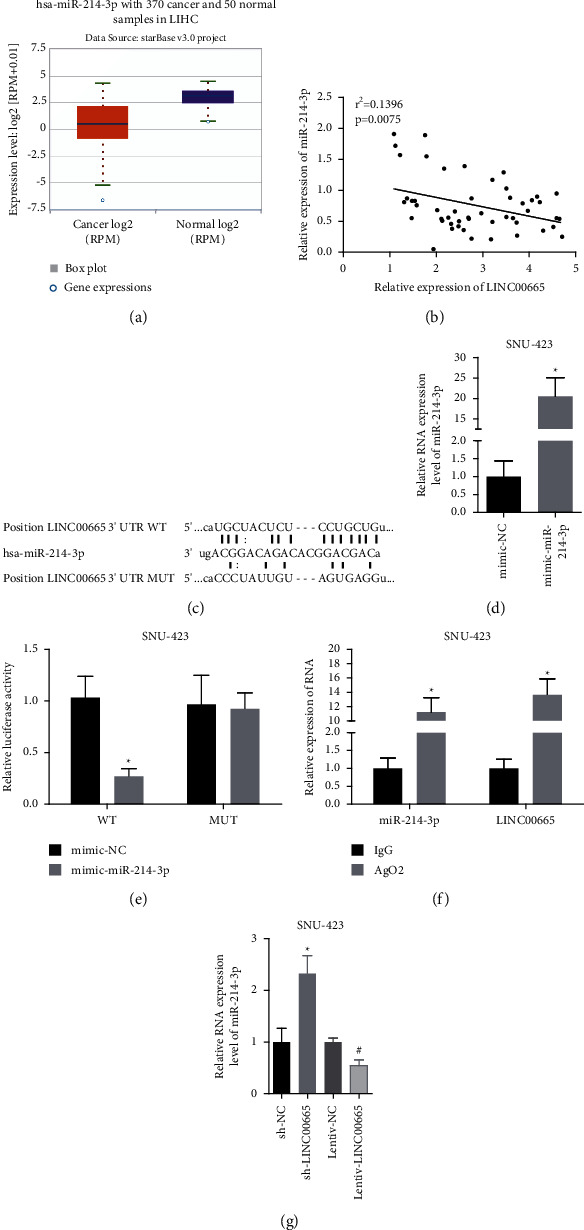
LINC00665 targeted miR-214-3p. (a) starBase database showed that miR-214-3p expression in HCC tissues was declined. (b) Pearson analysis of the association between the expression levels of LINC00665 and miR-214-3p in 50 HCC tissues. (c) miR-214-3p expression was measured by qRT-PCR assay. (d) Binding sites of LINC00665 and miR-214-3p. (e, f) Luciferase gene reporter and RIP assay were applied to assess the crosstalk between LINC00665 and miR-214-3p. (g) miR-214-3p expression was measured by qRT-PCR assay in sh-LINC00665/lentiv-LINC00665-treated SNU-423 cells (*n* = 3, *∗p* < 0.05).

Next, we explored the relationship between LINC00665 and miR-214-3p in HCC SNU-423 cells. From the starBase database, we found that miR-214-3p level was decreased in HCC tissues as compared with the normal tissues (Figure 3(a)). The Pearson correlation analysis showed that LINC00665 level was negatively associated with miR-214-3p level in 50 HCC tissues (Figure 3(b)). Then, we determined whether LINC00665 could target miR-214-3p. miR-214-3p level was elevated when the cells were transfected with mimic-miR-214-3p (Figure 3(c)). The putative binding sites of LINC00665 and miR-214-3p are shown in Figure 3(d). Overexpression of miR-214-3p resulted in a reduction in the luciferase activity, whereas this was abolished when the mutation was made between the binding sites (Figure 3(e)). Also, the RIP assay showed that LINC00665 and miR-214-3p could be enriched in Ago2 antibody (Figure 3(f)); and LINC00665 overexpression decreased miR-214-3p level in SNU-423 cells and vice versa (Figure 3(g)). These findings suggested that LINC00665 could target and decrease miR-214-3p expression in HCC.

### 3.4. LINC00665 Promotes HCC Viability and Aerobic Glycolysis via Decreasing of miR-214-3p Expression

**Figure 4:**
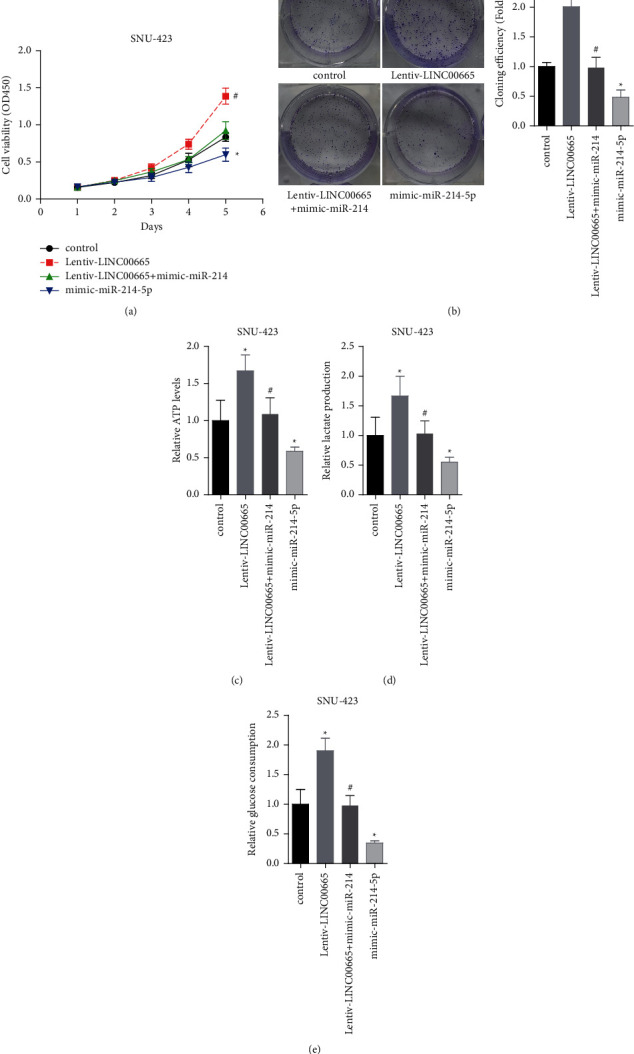
LINC00665 enhanced HCC viability and aerobic glycolysis via decreasing miR-214-3p expression. (a, b) LINC00665/miR-214-3p effect on cell viability was determined using CCK-8 and colony formation assay. (c–e) LINC00665/miR-214-3p effects on the ATP levels, lactate section, and glucose consumption were tested in SNU-423 (*n* = 3, *∗p* < 0.05 versus control group; #*p* < 0.05 versus lentiv-LINC00665 group).

We then explored whether miR-214-3p was involved in LINC00665-mediated HCC progression *in vivo*. The results demonstrated that miR-214-3p overexpression apparently inhibited cell viability and colony formation ability as compared with the control group (Figures 4(a) and 4(b)). Also, the enhancements in cell viability and colony formation ability induced by LINC00665 were decreased when miR-214-3p was overexpressed in SNU-423 cells (Figures 4(a) and 4(b)). In addition, miR-214-3p weakened LINC00665 role in promoting aerobic glycolysis glucose with decreased levels of ATP, lactate section, and glucose consumption (Figures 4(c)–4(e)). These results demonstrated that LINC00665 promoted HCC cell viability and aerobic glycolysis via decreasing miR-214-3p expression.

### 3.5. LINC00665 Promotes HCC Viability and Aerobic Glycolysis by Targeting miR-214-3p/MAPK1

**Figure 5:**
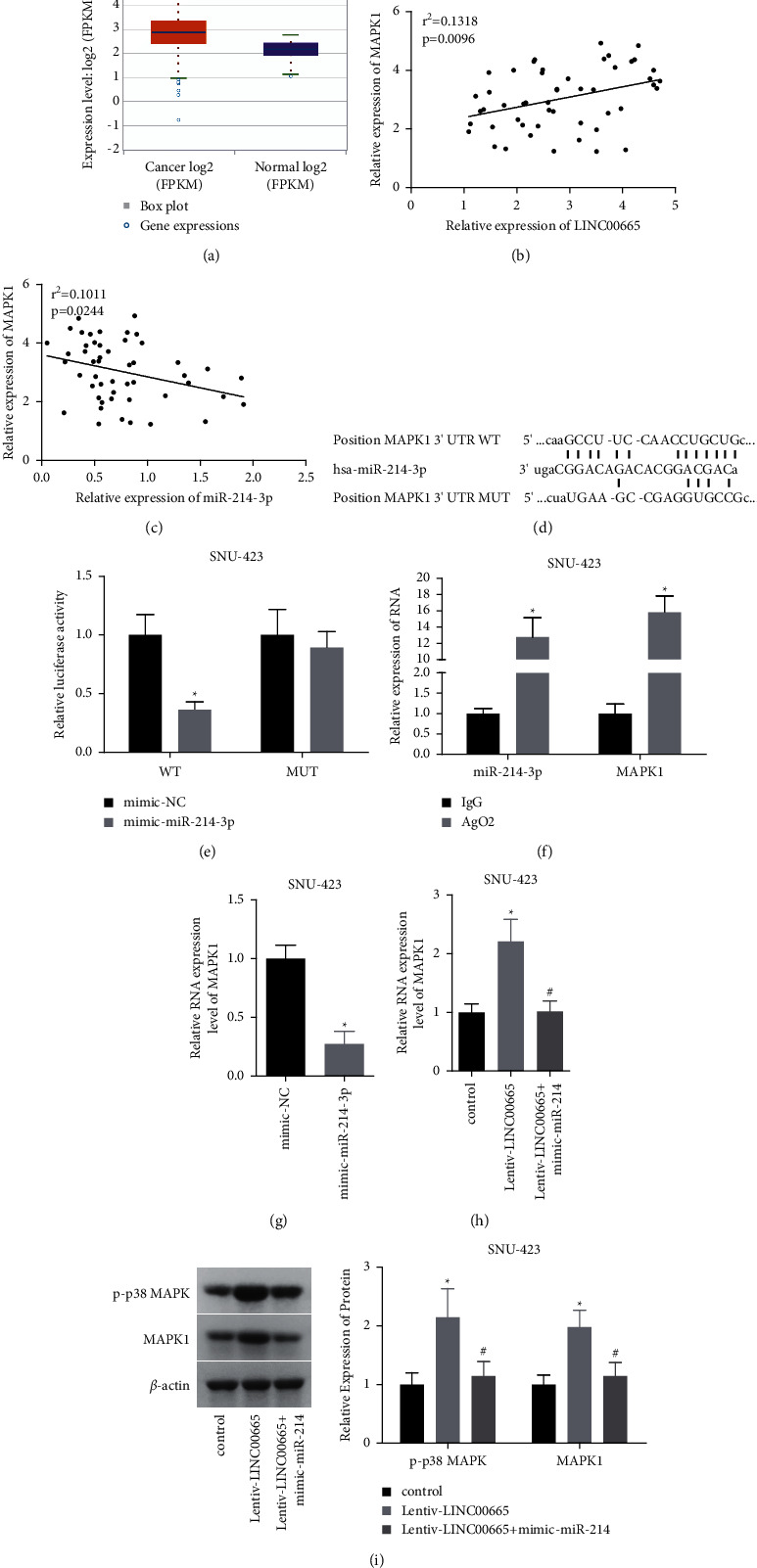
LINC00665 targeted miR-214-3p/MAPK1. (a) starBase database showed that MAPK1 was overexpressed in HCC tissues. (b, c) Pearson analysis of the association between the expression levels of LINC00665 and miR-214-3p in 50 HCC tissues. (d) Binding sites of miR-214-3p and MAPK1. (e, f) Luciferase gene reporter and RIP assay were applied to assess the crosstalk between LINC00665 and miR-214-3p. (g, h) MAPK1 expression in SNU-423 cells was measured by qRT-PCR assay. (i) The expressions of p-p38 MAPK and MAPK1 were determined by western blotting (*n* = 3, *∗p* < 0.05 versus control group; #*p* < 0.05 versus lentiv-LINC00665 group).

The starBase database showed that MAPK1 expression was increased in HCC tissues as compared with the normal tissues (Figure 5(a)). The Pearson correlation analysis showed that miR-214-3p level was positively associated with LINC00665 level in 50 HCC tissues (Figure 5(b)) and negatively linked to MAPK1 level (Figure 5(c)). To further disclose the mechanism by which LINC00665 promoted HCC viability and aerobic glycolysis, we then explored the interaction between miR-214-3p and MAPK1. The binding sites between miR-214-3p and MAPK1 are displayed in Figure 5(d). Overexpression of miR-214-3p decreased the luciferase activity of MAKK1-WT vector (Figure 5(e)); and the RIP assay showed that MAPK1 and miR-214-3p could be enriched in anti-Ago2 antibody (Figure 5(f)). Moreover, miR-214-3p decreased MAPK1 expression (Figure 5(g)) and rescued the increases in p-p38 MAPK and MAPK1 expressions induced by LINC00665 overexpression (Figures 5(h) and 5(i)). These results indicated that LINC00665 increased MAPK1 expression via sponging miR-214-3p.

**Figure 6:**
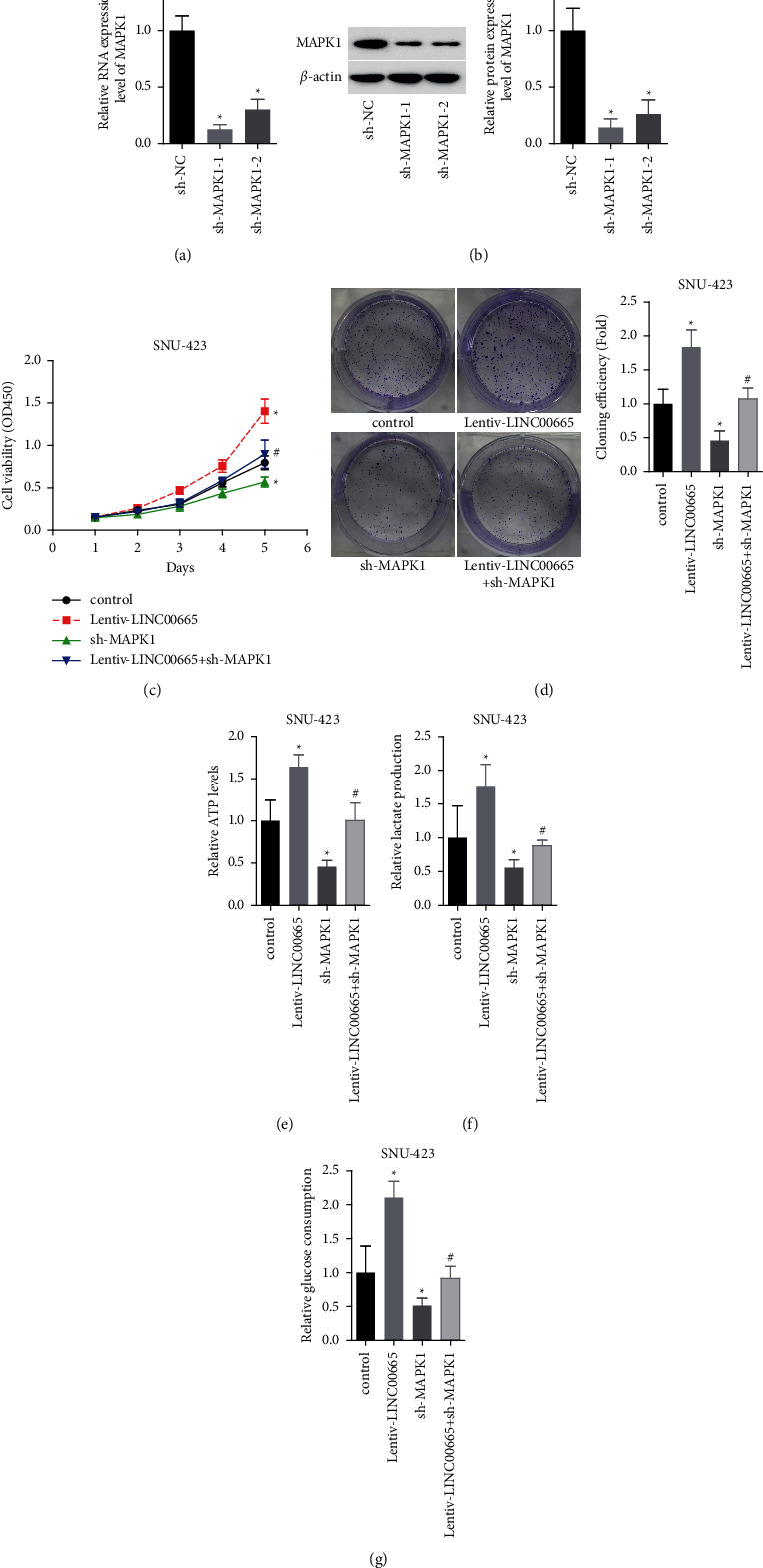
LINC00665 enhanced HCC viability and aerobic glycolysis via upregulation of MAPK1 expression. (a, b) qRT-PCR and western blotting were applied to detect MAPK1 expression (*n* = 3, *∗p* < 0.05). (c, d) LINC00665/MAPK1 effect on cell viability was determined using CCK-8 and colony formation assay. ((e–g)) LINC00665/MAPK1 effects on the ATP levels, lactate section, and glucose consumption were tested in SNU-423 (*n* = 3, *∗p* < 0.05 versus control group; #*p* < 0.05 versus lentiv-LINC00665 group).

Furthermore, we assessed MAPK1 role in LINC00665-mediated HCC progression. MAPK1 expression was significantly decreased when cells were infected with sh-MAPK1-1 and sh-MAPK1-2, and sh-MAPK1-1 showed the highest knockdown efficiency and hence was used in the following experiments (Figures 6(a) and 6(b)). Downregulation of MAPK1 markedly rescued the enhancements in cell viability (Figure 6(c)), colony formation (Figure 6(d)), and the increases in the levels of ATP, lactate section, and glucose consumption (Figures 6(e)–6(g)) induced by LINC00665 overexpression. These findings suggested that LINC00665 promoted cell viability and aerobic glycolysis by targeting miR-214-3p/MAPK1 in HCC.

### 3.6. Knockdown of LINC00665 Represses Tumor Formation In Vivo

**Figure 7:**
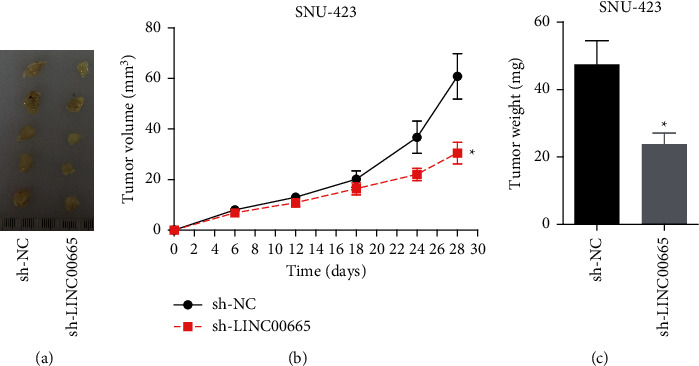
Knockdown of LINC00665 repressed tumor formation *in vivo.* (a) Tumor morphology of each group. (b) Tumor volume growth curve and (c) weight were assessed in sh-NC and sh-LINC00665 groups (*n* = 5/group, *∗p* < 0.05).

Also, the *in vivo* assay was performed to assess the role of LINC00665 in tumor formation. Compared with the sh-NC group, both of the tumor volume and weight in sh-LINC00665 group were decreased (Figures 7(a)–7(c)), indicating that knockdown of LINC00665 repressed tumor formation *in vivo*.

## 4. Discussion

Here, we first revealed the role of LINC00665/miR-214-3p/MAPK1 in modulating cell growth and aerobic glycolysis in HCC. The results demonstrated that LINC00665 was overexpressed in HCC, which accelerated cell growth and migration and triggered aerobic glycolysis through sponging miR-214-3p to increase MAPK1 expression.

Up to now, the oncogenic role of LINC00665 in several kinds of cancer has been disclosed through different mechanisms [15, 24–26]. For example, Zhou et al. [27] reported that LINC00665 was highly expressed in breast cancer tissues and cells, and the high expression of LINC00665 was linked to poor prognosis; deletion of LINC00665 attenuated the migration and invasion ability of breast cancer cells and inhibited epithelial-mesenchymal transition (EMT). Zhao et al. [17] found that LINC00665 was significantly upregulated in colorectal cancer tissues, which was negatively correlated with miR-9-5p expression but positively associated with ATF1 expression; and LINC00665 overexpression resulted in apparent enhancements in cell proliferation, migration, and invasiveness and reduced cell apoptosis by targeting miR-9-5p to increase ATF1 expression in colorectal cancer. In gastric cancer, works by Yang et al. [16] revealed that loss of LINC00665 repressed AGS and BGC-823 cell survival and cell expansion through inactivating the Wnt signaling pathway. Qi et al. [26] found that LINC00665 promoted cell tumorigenesis by modulating miR-149-3p/RNF2 axis in gastric cancer. In HCC, Shan and Li [19] reported that LINC00665 was overexpressed in HCC cells and tissue samples, which associated with poor prognosis of HCC patients; and LINC00665 depletion inhibited viability and induced apoptosis and autophagy through targeting miR-186-5p/MAP4K3 axis. Also, Ding et al. [28] found that the high expression of LINC00665 associated with poor outcome in HCC patients, and LINC00665 activated NF-kappaB signaling through blocking ubiquitin/proteasome-dependent degradation of PKR, leading to HCC growth. Consistently, we observed that the expression of LINC00665 was elevated in HCC tissues, and high level of LINC00665 predicted poor prognosis in patients with HCC. In addition, overexpression of LINC00665 promoted HCC cell growth, migration, invasion, and aerobic glycolysis with increased levels of ATP, glucose consumption, and lactate secretion, and depletion of LINC00665 caused opposite results. Aerobic glycolysis is a characteristic of cancer cells, through which the cancer cells can evade anoikis and even acquire growth and metastasis capacities [29, 30]. Thus, this study revealed a potential mechanism that LINC00665 promoted HCC growth and migration through inducing aerobic glycolysis.

In addition, we found that the expression of LINC00665 was negatively associated with miR-214-3p expression in HCC tissues, suggesting that there might be a link between LINC00665 and miR-214. The following assays (luciferase gene reporter and RIP assay) confirmed that LINC00665 could target miR-214-3p and decreased its expression. Contrary to the role of LINC00665, the *in vitro* assays showed that overexpression of miR-214-3p weakened HCC cell growth viability and inhibited aerobic glycolysis with decreased levels of ATP, glucose consumption, and lactate secretion. Consistently, miR-214-3p was also reported to be lowly expressed in cancers and inhibited cell growth in endometrial cancer [31], breast cancer [32], osteosarcoma [33], and lung cancer [32]. In HCC, Li et al.'s work [20] showed that miR-214-3p was downregulated in HCC, and miR-214-3p precursor impaired cell growth, induced cell G1 phase arrest, and promoted cell apoptosis in HUH-7 and HepG2 cells, suggesting that miR-214-3p inhibited HCC progression. In the present study, we first revealed the role of miR-214-3p in aerobic glycolysis. Also, we observed that miR-214-3p reversed LINC00665-mediated cell viability and aerobic glycolysis enhancements, demonstrating that LINC00665 promoted HCC progression through decreasing miR-214-3p level.

Moreover, our results illustrated that MAPK1 was highly expressed in HCC tissues and positively associated with LINC00665 expression but negatively associated with miR-214-3p level in HCC tissues. Further exploration showed that LINC00665 increased the expression of MAPK1 and its phosphorylation level through sponging miR-214-3p. MAPK1 has been found to be overexpressed in follicular lymphoma [34] and its activation apparently triggers the EMT process of cervical cancer [35]. Herein, we found that silencing of MAPK1 suppressed cell viability and aerobic glycolysis in HCC cells and rescued the enhancement of cell viability and aerobic glycolysis induced by LINC00665, indicating that LINC00665 promoted HCC progression through upregulation of MAPK1.

## 5. Conclusion

In summary, this study demonstrated that LINC00665 and MAPK1 were overexpressed in HCC tissues, while miR-214-3p expression was declined. Overexpression of LINC00665 accelerated cell growth and induced Warburg effect through sponging miR-214-3p to increase MAPK1 expression in HCC. This study first points out that aerobic glycolysis pathway is a potential mechanism by which LINC00665 promotes the malignant progression of HCC.

## Figures and Tables

**Table 1:** Primer sequences.

Gene	Forward (5′-3′)	Reverse (5′-3′)
MAPK1	CAGTTCTTGACCCCTGGTCC	TACATACTGCCGCAGGTCAC
LINC00665	AGCACCCCTAGTGTCAGTCA	TGGTCTCTAGGGAGGCAGAA
*β*-Actin	CTTCGCGGGCGACGAT	CCACATAGGAATCCTTCTGACC

## Data Availability

The data and materials used to support the findings of this study are included within the published article.

## References

[1] Sung H., Ferlay J., Siegel R. L. (2021). Global cancer statistics 2020: GLOBOCAN estimates of incidence and mortality worldwide for 36 cancers in 185 countries. *CA: a Cancer Journal for Clinicians*.

[2] Liang Y., Zhang D., Zheng T. (2020). lncRNA-SOX2OT promotes hepatocellular carcinoma invasion and metastasis through miR-122-5p-mediated activation of PKM2. *Oncogenesis*.

[3] Venook A. P., Papandreou C., Furuse J., de Guevara L. L. (2010). The incidence and epidemiology of hepatocellular carcinoma: a global and regional perspective. *The Oncologist*.

[4] Lunt S. Y., Vander Heiden M. G. (2011). Aerobic glycolysis: meeting the metabolic requirements of cell proliferation. *Annual Review of Cell and Developmental Biology*.

[5] Schwartz L., Supuran C., Alfarouk K. (2017). The warburg effect and the hallmarks of cancer. *Anti-Cancer Agents in Medicinal Chemistry*.

[6] Vaupel P., Schmidberger H., Mayer A. (2019). The warburg effect: essential part of metabolic reprogramming and central contributor to cancer progression. *International Journal of Radiation Biology*.

[7] Xu F., Yan J. J., Gan Y. (2019). miR-885-5p negatively regulates warburg effect by silencing hexokinase 2 in liver cancer. *Molecular Therapy Nucleic Acids*.

[8] Zhang X., Guo J., Jabbarzadeh Kaboli P. (2020). Analysis of key genes regulating the warburg effect in patients with gastrointestinal cancers and selective inhibition of this metabolic pathway in liver cancer cells. *OncoTargets and Therapy*.

[9] Volders P. J., Verheggen K., Menschaert G. (2015). An update on LNCipedia: a database for annotated human lncRNA sequences. *Nucleic Acids Research*.

[10] Xue C., Chen C., Gu X., Li L. (2021). Progress and assessment of lncRNA DGCR5 in malignant phenotype and immune infiltration of human cancers. *American Journal of Cancer Research*.

[11] Qi X., Lin Y., Liu X., Chen J., Shen B. (2020). Biomarker discovery for the carcinogenic heterogeneity between colon and rectal cancers based on lncRNA-associated ceRNA network analysis. *Frontiers in Oncology*.

[12] Qi X., Zhang D. H., Wu N., Xiao J. H., Wang X., Ma W. (2015). ceRNA in cancer: possible functions and clinical implications. *Journal of Medical Genetics*.

[13] Wang D., Liu K., Chen E. (2020). LINC00511 promotes proliferation and invasion by sponging miR-515-5p in gastric cancer. *Cellular & Molecular Biology Letters*.

[14] Lv M., Mao Q., Li J., Qiao J., Chen X., Luo S. (2020). Knockdown of LINC00665 inhibits proliferation and invasion of breast cancer via competitive binding of miR-3619-5p and inhibition of catenin beta 1. *Cellular & Molecular Biology Letters*.

[15] Chen W., Yu Z., Huang W., Yang Y., Wang F., Huang H. (2020). LncRNA LINC00665 promotes prostate cancer progression via miR-1224-5p/SND1 axis. *OncoTargets and Therapy*.

[16] Yang B., Bai Q., Chen H., Su K., Gao C. (2020). LINC00665 induces gastric cancer progression through activating Wnt signaling pathway. *Journal of Cellular Biochemistry*.

[17] Zhao X., Weng W., Long Y., Pan W., Li Z., Sun F. (2020). LINC00665/miR-9-5p/ATF1 is a novel axis involved in the progression of colorectal cancer. *Human Cell*.

[18] Xu D., Song Q., Liu Y. (2021). LINC00665 promotes ovarian cancer progression through regulating the miRNA-34a-5p/E2F3 axis. *Journal of Cancer*.

[19] Shan Y., Li P. (2019). Long intergenic non-protein coding RNA 665 regulates viability, apoptosis, and autophagy via the MiR-186-5p/MAP4K3 axis in hepatocellular carcinoma. *Yonsei Medical Journal*.

[20] Li Y., Li Y., Chen Y. (2017). MicroRNA-214-3p inhibits proliferation and cell cycle progression by targeting MELK in hepatocellular carcinoma and correlates cancer prognosis. *Cancer Cell International*.

[21] Hao J. F., Chen P., Li H. Y., Li Y. J., Zhang Y. L. (2020). Effects of LncRNA HCP5/miR-214-3p/MAPK1 molecular network on renal cell carcinoma Cells. *Cancer Management and Research*.

[22] Livak K. J., Schmittgen T. D. (2001). Analysis of relative gene expression data using real-time quantitative PCR and the 2(-delta delta C(T)) Method. *Methods*.

[23] Zhou Q., Zhang W., Wang Z., Liu S. (2019). Long non-coding RNA PTTG3P functions as an oncogene by sponging miR-383 and up-regulating CCND1 and PARP2 in hepatocellular carcinoma. *BMC Cancer*.

[24] Dai H., Sheng X., Sha R. (2021). Linc00665 can predict the response to cisplatin-paclitaxel neoadjuvant chemotherapy for breast cancer patients. *Frontiers in Oncology*.

[25] Ji W., Diao Y. L., Qiu Y. R., Ge J., Cao X. C., Yu Y. (2020). LINC00665 promotes breast cancer progression through regulation of the miR-379-5p/LIN28B axis. *Cell Death & Disease*.

[26] Qi H., Xiao Z., Wang Y. (2019). Long non-coding RNA LINC00665 gastric cancer tumorigenesis by regulation miR-149-3p/RNF2 axis. *OncoTargets and Therapy*.

[27] Zhou J. L., Zou L., Zhu T. (2020). Long non-coding RNA LINC00665 promotes metastasis of breast cancer cells by triggering EMT. *European Review for Medical and Pharmacological Sciences*.

[28] Ding J., Zhao J., Huan L. (2020). Inflammation-Induced long intergenic noncoding RNA (LINC00665) Increases malignancy through activating the double-stranded RNA-activated protein kinase/nuclear factor kappa B pathway in hepatocellular carcinoma. *Hepatology*.

[29] Lu J., Tan M., Cai Q. (2015). The warburg effect in tumor progression: mitochondrial oxidative metabolism as an anti-metastasis mechanism. *Cancer Letters*.

[30] Vander Heiden M. G., Cantley L. C., Thompson C. B. (2009). Understanding the warburg effect: the metabolic requirements of cell proliferation. *Science*.

[31] Fang Y. Y., Tan M. R., Zhou J. (2019). miR-214-3p inhibits epithelial-to-mesenchymal transition and metastasis of endometrial cancer cells by targeting TWIST1. *OncoTargets and Therapy*.

[32] Han L. C., Wang H., Niu F. L., Yan J. Y., Cai H. F. (2019). Effect miR-214-3p on proliferation and apoptosis of breast cancer cells by targeting survivin protein. *European Review for Medical and Pharmacological Sciences*.

[33] Yang Y., Li Z., Yuan H. (2019). Reciprocal regulatory mechanism between miR-214-3p and FGFR1 in FGFR1-amplified lung cancer. *Oncogenesis*.

[34] Wang W., Corrigan-Cummins M., Hudson J. (2012). MicroRNA profiling of follicular lymphoma identifies microRNAs related to cell proliferation and tumor response. *Haematologica*.

[35] Du L., Rao G., Wang H. (2013). CD44-positive cancer stem cells expressing cellular prion protein contribute to metastatic capacity in colorectal cancer. *Cancer Research*.

